# Perceptions of Practitioners on Telehealth and App Use for Smoking Cessation and COPD Care—An Exploratory Study

**DOI:** 10.3390/medicina56120698

**Published:** 2020-12-15

**Authors:** Daniela Haluza, Michaela Saustingl, Kseniya Halavina

**Affiliations:** Department of Environmental Health, Center for Public Health, Medical University of Vienna, 1090 Vienna, Austria; n1309536@students.meduniwien.ac.at (M.S.); n1242820@students.meduniwien.ac.at (K.H.)

**Keywords:** health information technology, health behavior, influencing factors, online resources, lung disease, smoking cessation therapy

## Abstract

*Background and objectives:* With the digitalization of modern healthcare delivery, digital media adoption in clinical practice is increasing. Also, healthcare professionals are more and more confronted with patients using smartphone-based health applications (apps). This exploratory study aimed at surveying perceptions on such apps in the context of lung health among a cross section of Austrian practitioners involved in pulmonary care. *Materials and Methods:* The online questionnaire in German assessed socio-demographic characteristics, telehealth readiness as well as opinions on smoke-free and COPD (chronic obstructive pulmonary disease) apps. We used descriptive statistics to report the finding. *Results:* We received valid responses from 55 participants (mean age 52.3 years, 69.1% males). Telehealth readiness was medium, indicating existence of certain barriers adversely impacting telehealth use. As for apps targeting smoking cessation and COPD, respondents indicated high relevance for visualization aspects for patients and control/overview features for the treating doctors. Only 40% of participants indicated that they would recommend a COPD app to an older patient. *Conclusions:* In smoking cessation therapy, doctors commonly adhere to the “5 A’s”: Ask, Advise, Assess, Assist, and Arrange. We suggest adding “App” as sixth A, assuming that in patient follow-up most of the other A’s could also be supported or even replaced by app features in the challenging task to tackle smoking-associated non-communicable diseases.

## 1. Introduction

Telehealth, or telemedicine, refers to the use of IT for remote personalized care, treatment, and prevention [1,2]. Digital health strategies explicitly link the user-driven development of healthcare provision to smartphone technology, with the installation of various third-party applications (apps) being among the most abundantly used smartphone features [3]. Global smartphone use and mobile internet access via these devices are very high these days. As an example, smartphone penetration grew to 97% in Austria in 2019, with 29% having installed eleven to 20 apps on their smartphones [4].

Smoking tobacco contributes to 12% of deaths globally [5]. According to latest Eurostat figures, the proportion of daily cigarette smokers in Austria is 24%, placing it sixth among the EU member states [6]. Although a multifactorial disease, smoking is the leading behavioral risk factors for COPD (chronic obstructive pulmonary disease), a common obstructive disease of the lower airways [7,8]. In 2015, 68% of US adult smokers wanted to quit smoking, 57% had been advised by a health professional to quit, and 31% sought counseling and/or medication for cessation [9]. Considering the large amount of smokers, these percentages translate into a challenge for the healthcare system. The literature strongly suggests that doctors play a critical role in successful smoking cessation [10]. There is a high penetration of smartphone use, and there are documented benefits for smoke quitting patients when keeping regular contact to their doctor. Therefore, apps for facilitating interaction and follow-up significantly increase the short- and long-term success of smoke cessation treatments [10,11,12,13,14].

Pifarré et al. estimated for the Catalan Public Health System that the smoke quitting app TControl could save healthcare costs of about 400 million € per decade [15]. Studies on digital media found that physicians acknowledge potential benefits on patient and system level and are especially interested in tools supporting their clinical work [16,17,18]. Commonly expressed concerns relate to quantity of data, patient motivation to utilize equipment, and distortions of the doctor-patient relationship. Whereas adoption of more elaborate telehealth technologies by rural primary care clinicians may be influenced by equipment implementation resources and payment, smartphone apps are available at low or no costs.

Doctors are key facilitators for app adoption and have to provide patient education and guidance through the app jungle [10]. So, strategic planning and targeted telehealth adoption strategies depends on knowing their respective opinions and practices [16,17,18]. So far, little evidence is available for the Austrian situation in this respect. Based on these considerations, our exploratory study aimed at identifying the prevailing perceptions regarding telehealth and health app use in the context of lung health. We surveyed a purposive, non-probabilistic sample of Austrian practitioners to identify (i) perceived importance of health app features and benefits, (ii) telehealth readiness, and (iii) scenario-based preferences for smoke-free and COPD apps.

## 2. Methods

### 2.1. Study Design

We created a survey tool assessing health app use and telehealth readiness in German based on a questionnaire validated in prior research [18,19,20,21,22,23,24,25]. In addition, three pragmatic vignettes describing potential real-life situations as widely used in research elucidating intended actions and perceptions of healthcare personnel were included in the survey [17,21,22,23,24,26]. Ten lay persons were invited to pilot-test a preliminary paper-pencil version in order to ensure comprehensibility and readability. The adapted online version was again pilot-tested, and the final online survey was accessible barrier-free via the web-based survey tool SoSci Survey from 19 September 2019 to 21 January 2020 [27].

As we strived at surveying doctors treating patients with lung problems, we retrieved an email list of registered Austrian pulmonologists (*n* = 486 in 2019) from the Austrian Medical Chamber as well as Austrian hospitals and healthcare providers [28]. We sent the non-personalized link to the online questionnaire to potential survey participants via email invitations and newsletter of the Austrian Respiratory Society [29]. The cover page informed participants about the study aim, and their consent was implicitly obtained when completing the survey. We did not offer incentives for study participation. The survey protocol was approved by the institutional ethical committee of the Medical University of Vienna, Austria, on 1 February 2018, and the study was conducted following the ethical standards laid down in the Declaration of Helsinki. The questionnaire in German is available from the author upon request.

### 2.2. Study Questionnaire

German-speaking, Austrian practitioners treating patients with lung problems, as evaluated using a respective filter question, were eligible for participation. The online survey collected data on socio-demographic characteristics age (in years), gender (male, female), specialization (pulmonologist, other e.g., general practitioner, internist, cardiologist), residence (Vienna, Upper Austria, other Austrian counties), geographical area (rural, urban), statutory health insurance physician (i.e., having a contract with a statutory health insurance, yes, no), and smartphone and private health app use (both: yes, no).

Participants were further asked to rate six potential benefits (yes/no) of app use. Location-independent access to health services, higher quality of healthcare, higher efficiency in healthcare resource allocation, reduced healthcare costs, higher efficiency in medical consultation, and improved doctor-patient relationship. Study subjects were invited to indicate which features are important when using a health app, with the options: free use, easy to use, clarity, speed, facilitated clinical work, recommendation from colleagues, proof of quality, facilitated communication with patients, electronic collection of health information, and management of health data.

We asked participants to indicate their agreement with the following five statements using on a 5-point Likert scale ranging from disagree = 1 to agree = 5: “The use of medical apps during doctor-patient contact disturbs the relationship to the patient.”, “I only use medical apps privately, not during my medical work.”, “I could no longer imagine everyday medical practice without the use of medical apps.”, “In an emergency, I give patients the opportunity to contact me by phone.”, and “I give patients the opportunity to contact me via internet-based means of communication (email, online form, etc.).”.

We asked participants to answer the question “What would you like to improve with telehealth services?” using the three yes/no options service and treatment quality, internal processes, and turnover. The two items “How would you feel about offering telehealth service to COPD patients?” and “How would you feel about COPD patients paying for telehealth services, at least a certain deductible?” collected respective opinions on a 5-point Likert scale (from disagree = 1 to agree = 5).

A further part of the survey contained the commonly used practitioner telehealth readiness assessment tool (PRAT) in German validated in prior research [18,21,30,31,32,33]. The PRAT is a scale of 17 items using a 5-point Likert scale ranging from disagree = 1 to agree = 5 to assess a broad range of aspects of readiness for telehealth. These include dissatisfaction with the status quo, expectation of change, awareness and perceived benefits and barrier, adequate technical infrastructure and soft skills. A summed score thus indicates readiness for telehealth.

The last part of the questionnaire consisted of the three following scenarios, evaluating the key features of smoke-free apps and COPD apps as reported in the pertinent literature [8,10,11,12,13,14,34]. For the scenarios, it is of relevance that COPD is classified into stages, with COPD Gold I being the mildest and COPD Gold IV being the severest one, mirroring the classical symptoms pulmonary hypertension and pulmonary heart disease, shortness of breath, and reduced physical performance [8].

Scenario 1. *Smoke-free app* collects participants’ opinions on smoke-free app features from the perspective of the patient (5 items) and treating doctor (3 items) on a 5-point Likert scale ranging from unimportant = 1 to important = 5.

“A 26-year-old woman visits your private practice/the ambulance. She tells you that she has been smoking for six years, but now wants to stop because she wants to get pregnant. You recently heard about a new smoke-free app that provides interactive support for quitting smoking. You recommend this to your patient. In your opinion, how important are the following functions of the smoke-free app for your patient?” and “In your opinion, how important are the following functions of the smoke-free app for you as a treating doctor?”.

Scenario 2. *COPD app* collects opinions on the functions of a COPD app from the perspective of the patient (7 items) and the treating doctor (3 items) on a 5-point Likert scale ranging from unimportant = 1 to important = 5.

“A 64-year-old patient with pretreated, moderate COPD Gold II visits your private practice/the ambulance for the first time. You recently heard about a new mobile app to support the treatment of COPD. You recommend this to your patient. In your opinion, how important are the following functions of this COPD app for the patient?” and “In your opinion, how important are the following functions of this COPD app for you as a treating doctor?”

Scenario 3. *COPD app recommendation* collects opinions on counseling practice:

“A 75-year-old patient visits your private practice/the ambulance for a check-up. He has moderate COPD Gold III. You recently heard of a mobile app to support the treatment of COPD. The patient tells you that he has a smartphone. Would you normally recommend using this app to this patient?”, with the answer options yes, no, and undecided.”

### 2.3. Statistical Data Analysis

We conducted all reported statistical analyses using SPSS Statistics for Windows, Version 26.0 (Armonk, NY, USA, IBM Corp.). Only fully completed surveys were included in the analysis. We descriptively summarized collected data to present categorical data as absolute and relative frequencies, and continuous data as mean, standard deviation (SD). Rounded figures are presented in the text to increase readability. We calculated the aggregated telehealth readiness score (i.e., mean score of the 17 items of the PRAT) with higher ratings indicated higher degree of agreement. To determine the internal consistency of the scores, we calculated Cronbach’s alpha (alpha).

## 3. Results

### 3.1. Study Population

The link to the online survey was accessed 217 times; 78 participants started and 55 of those fully completed the survey (25.4% completion rate). Average age of study participants was 52.3 (SD 10.6, range 31–76 years), 69.1% were male, 34.6% lived in Vienna, 78.2% indicated they live in an urban area, and almost all participants used smartphones (96.4%, Table 1).

### 3.2. Perceptions on Health Apps

Location-independent access to health services was the most important benefit of health apps (54.5%), followed by increased efficiency in medical consultation (40.0%) as well as in healthcare resource allocation (29.1%). Further ranks were taken by higher quality of healthcare (27.3%), improved knowledge of the patient, and improved doctor-patient relationship (both 12.7%). Notably, 14.5% of participants perceived that there would be no benefits of health app use.

The following app features yielded highest ratings (all > mean 4.0, Table 2): easy to use, clarity, facilitated clinical work, speed, and proof of quality.

Participants rather agreed that they would only use apps privately, not during clinical work (mean 3.3, SD 1.4), whereas they showed a tendency to avoid providing patients with the opportunity to contact them by phone (mean 3.0, SD 1.5 or mean 2.8, SD 1.6). Participants would rather not miss apps in everyday medical practice (mean 2.0, SD 1.2).

Study subjects indicated that telehealth would be suitable to improve service and treatment quality (81.8%), internal processes (50.9%), and turnover (18.2%). Participants showed interest in offering telehealth services to COPD patients (mean 3.0, SD 1.2) and indicated that patients should pay for these services (copay or deductible; mean 3.4, SD 1.4).

### 3.3. Readiness Assessment for Telehealth

With an average score of 3.0 (SD 0.6, range 1.5 to 4.4), i.e., 59.9% of the maximum reachable score of 5, the telehealth readiness score was of medium size (alpha = 0.832, Table 3). We found the highest approval for the items “I have respect for others in the telehealth team.” (mean 4.0, SD 1.1), “I have a sense of curiosity about the influences of telehealth on improving the delivery of health care.” (mean 3.9, SD 1.0), and “I have firsthand experience of the negative effects of isolation from healthcare services.” (mean 3.8, SD 1.2). Lowest agreement was yielded for “I have telehealth reimbursement plans in place.” (mean 1.7, SD 1.0).

### 3.4. Scenarios on Lung Health App Use

Opinions on scenarios on app use are presented in Table 4. In Scenario 1. *Smoke-free app*, we found highest ratings for the option “Representation of the positive effect on the body over time” (mean 4.4, SD 1.0), with viewing the patient´s progress electronically as most important feature (mean 3.6 SD 1.3). In Scenario 2. *COPD app*, we found highest ratings for “The app shows the patient how to breathe and inhale correctly with videos and breathing exercises.” (mean 4.2, SD 1.2) with viewing the results of the regular COPD assessment tests electronically as most important feature (mean 3.6, SD 1.2). In Scenario 3. *COPD app recommendation*, 40.0% of the participants would recommend a COPD app to the older patient, 25.5% would rather not, and 34.5% were undecided.

## 4. Discussion

In this online survey among a cross-section of Austrian practitioners, we identified perceptions on health app features and benefits, telehealth readiness, and scenarios on smoke-free and COPD apps.

Health apps aim at positively influencing the social, physical, and emotional well-being of users [30,35,36]. Patient-centered, disease-specific apps have the potential to empower patients while facilitate disease management and doctor-patient communication [3]. In the context of lung health, mobile apps for smoking cessation therapy and continuously monitoring symptoms in patients with COPD are a growing market that experienced a boom in the last decade [11,37]. Smoking cessation is beneficial to health at any age and also constitutes an important pillar of COPD treatment [8,38]. In a 2018 study, app characteristics were more relevant for the intention to use a cessation app than demographic characteristics such as age and level of education [39]. In clinical practice, satisfactory long-term outcomes depend on a personalized approach in the doctor-patient-app triangle, combining face-to-face counseling, pharmacotherapy, and (a) supportive app(s) with medical devices such as a carbon monoxide checker or a spirometer [12].

In accordance with recently published studies, prevalent smartphone and app use among study participants was high and corresponds to the ubiquitous digital media penetration in the general Austrian population [4,24,40]. Also, our findings confirmed prior knowledge that location-independent access to health services and increased efficiency in medical consultation were the top important benefit for health apps. Notably, nearly 30% of respondents rated healthcare resource allocation and higher quality of healthcare as a relevant advantage, highlighting the so far unexploited potential for app-based healthcare provision.

In our study, participants indicated their level of telehealth readiness by answering the PRAT, which captures the multidimensional concept of telehealth readiness [18,19,41]. Telehealth readiness in our current sample was of medium range, indicating that certain barriers exist that adversely impact telehealth use, which is in good accordance with observations reported from similar studies [18,19,20,41]. As suggested in the pertinent literature, this finding is speculatively caused by various reasons such as a still evolving digitalization process, lack of incentives for telehealth services, aging of the medical profession due to the baby boomer generation and being digital immigrants, technology skepticism among doctors in regard to telehealth, poor funding for telehealth provision, and assessment tool shortcomings [21,22,23,42].

As far as we know, this exploratory study is the first attempt to evaluate scenario-based views of Austrian practitioners on lung health apps. We chose two very common disease-centered apps, namely for smoke cessation and COPD therapy, assuming that smoke cessation is a prerequisite for both prevention and treatment of COPD. In both settings, the visualization aspect for the patient and the control and overview feature for the treating doctor were rated highest. This observation that provides a first insight into future in-depth research directions is supported by the previously published literature on health app adoption [15,34,36,37,43]. Earlier generations of smoking cessation apps did not provide tailored feedback, although users would prefer this feature [13]. Thus, interactive apps are more effective compared to static information apps on continuous smoking abstinence [14]. Also, gain-framed features could be more effective than loss-framed ones [34]. Peng et al. suggested using personalized apps with information sharing features so that patients can customize and control their health data [43].

In the year 2020, the coronavirus disease 2019 (COVID-19) pandemic caused by the severe acute respiratory syndrome coronavirus 2 (SARS-CoV-2) has changed life as we knew it, on a global-scale [44]. On the other hand, this international medical and socio-economic crisis is very likely to mark the dawn of a new era of healthcare delivery embracing telehealth [36]. To increase health app adoption, Vitacca et al. suggested involving end users, i.e., patients and doctors alike, in the development and implementation of disease management tools [45]. From a Public Health perspective, targeted education and adequate incentives and funding for both patients and healthcare providers could play a role in motivating continued use [43]. This fact is often neglected, which is especially frustrating for doctors [16,19]. Interestingly, whereas low and no cost health apps are commonly preferred by patients, in our study, participants did not rate free use of the apps among the top priorities [35]. Also, participants expected that telehealth could outstandingly improve service and treatment quality and internal processes, with their financial benefit being a somewhat indirect result of time saving and flexibility. Participants showed medium range interest in offering telehealth services to COPD patients, but indicated that patients should pay for these services, at least a certain deductible. Health insurers can increase smoking cessation by covering and promoting evidence-based treatments [9]. As highlighted by study participants, telehealth reimbursement plans are inadequate. In the challenging task to tackle smoking-associated non-communicable diseases, a so far lacking holistic approach is needed, balancing social and economic values with financial return for doctors covering time-consuming counseling on healthy behavior [18,19].

Research highlights that doctors should provide more than just advice to quit for maximizing smoking cessation rates, as smokers are more likely to quit when cessation medication and frequent counseling opportunities are combined [46]. This concept is well-known as the 5-As tobacco cessation treatment model: (1) ask patients about smoking at every visit, (2) advise all tobacco users to quit, (3) assess their willingness to quit, (4) assist their efforts with treatment and recommendations, and (5) arrange follow-up contacts to support cessation efforts. To adhere to the new age of digitization, this practical framework should be amended by (6) apply a mobile smoking cessation app, reflecting the doctor-patient-app triangle in a trustful doctor-patient relationship [24].

Successful intervention begins with the patient’s willingness to quit. Oliver et al. found that although most smokers (80%) own a smartphone, their experience with smoking cessation apps was rare (6%) [34]. The original smartphone function of serving as mobile communication devices has been expanded, as it is used as personal computer with capacious memories and large screens suitable for convenient app use. So, we suggest a modernized scheme, namely the “6 A’s”: Ask, Advise, Assess, Assist, Arrange, and App. With cessation apps advancing in user friendliness and applicability, a large number of the other A´s could also be supported or even replaced by app features in patient follow-up.

Regarding practical and theoretical implications of the main study findings, we did not expect that the majority of participants would rather abstain from recommending a COPD app to an older patient owning a smartphone. This finding could root in several factors, such as anticipating low health illiteracy, poor technology skills of digital immigrants with resulting low chances of adequate therapeutic success, similar own treatment experience, and expecting a disproportionate amount of time investment in patient follow-up [30,47]. In view of aging populations, this interesting observation warrants to further elucidate underlying barrier towards telehealth applications in older patients in in-depth mixed-methods studies.

Given our finding that certain barriers exist that adversely impact telehealth use, this exploratory study prepare the ground for future research, which should be refined in more complex research approaches using e.g., mixed-methods in a representative study population of doctors-and patients, nurses, and other healthcare stakeholders, also including other nationalities [48].

## 5. Limitations

Our study had limitations that should be kept in mind when interpreting our observations. As with all cross-sectional surveys, we were not able to evaluate trends in individual behavior over time. As we conducted an online study, we did not use incentives for participation, which could clearly increase response rates in follow-up studies. Although similar sample sizes are reported in related studies, the response rate was low, potentially mirroring the fact that health professionals are commonly considered as a hard to reach population, and [18,19,33,49,50,51]. Notably, we abstained from reporting results of inferential statistics, as, potentially due to the small sample size in the respective subgroups, age, gender, and specialization did not differ regarding the telehealth readiness score (data not shown).

We relied on participants’ self-reports, which introduced recall bias. The anonymous nature of the online survey in which there was no prior relationship with the potential recipients (i.e., the blind date of online surveys) did not allow for analyzing potential reasons for non-response. There is the possibility of unmeasured confounding associated with opinions on app and telehealth use such as lack of interest in the telehealth issue or even resistance to telehealth. Using a convenience sampling approach, we excluded non-health professionals as well as participants who did not read and understand German, limiting generalizability of the results and representativeness of our study population. However, we aimed at surveying a highly-educated academic homogenous study population of Austrian doctors.

As for the scenarios, we assumed that participants judged scenario validity based on the level of scenario realism rather than on its ability to answer predefined questions. We aimed at standardizing the context of the questions using pragmatic vignettes elucidating potential real-life actions.

## 6. Conclusions

The current exploratory study on digital media increased the understanding of doctors’ opinions and preferences in the context of lung health in Austria. In today’s digitalized world, patients are willing to adopt telehealth options, a trend that is very likely to be continued in the future, with the smartphone in everybody’s pocket. Far-sighted health policy and decision-making strategies should integrate apps and small monitoring devices as tools to substantially increase empowerment and health literacy in chronically ill patients as well as for health prevention. Notably, our study aimed at surveying Austrian doctors treating patients with lung problems. In Austria, as in other countries, smoking cessation and COPD treatment is very often done by non-pulmonologists, e.g., general practitioners, internists, cardiologists etc., as too few pulmonologists are available and waiting time for a doctor’s appointment are thus long, especially in rural areas. To increase effectiveness and efficiency in personal contacts in the doctor-patient-app triangle in all relevant medical fields, adequate funding for patient education and guidance through the somewhat overwhelming app jungle is a prerequisite.

This study was conducted just some weeks before the COVID-19 spread made the world stand still in March 2020. The pandemic has dramatically shown that preventing lung parenchyma from damage as from chronic smoke exposure is potentially lifesaving in case of a viral infection. Besides that, the global pandemic ushered in a new era of telehealth-based healthcare delivery. So, our results could be seen as a baseline for a further pre-post study comparing the awareness among doctors in regard to using apps for treating their patients from a distance.

## Figures and Tables

**Table 1:** Socio-demographic characteristics of the study population (*n* = 55).

		*n*	%
Specialization	Pulmonologist	42	76.4
	Other	13	23.6
Gender	Male	38	69.1
	Female	17	30.9
Residence	Upper Austria	11	20.0
	Vienna	19	34.6
	Other	10	18.2
Geographical area	Urban	43	78.2
	Rural	12	21.8
Statutory health insurance physician	No	23	41.8
	Yes	32	58.2
Smartphone use	Yes	53	96.4
	No	2	3.6
Private health app use	Yes	22	40.0
	No	33	60.0

**Table 2:** Ranking of importance of app features (*n* = 55).

	Mean	SD
Free use	3.91	1.22
Easy to use	4.78	0.63
Clarity	4.75	0.64
Speed	4.60	0.78
Facilitated clinical work	4.71	0.71
Recommendation from colleagues	3.44	1.07
Proof of quality	4.40	0.81
Facilitated communication with patients	3.36	1.32
Electronic collection of health information	3.09	1.27
Management of health data	3.00	1.09

SD: standard deviation.

**Table 3:** Readiness assessment for telehealth (*n* = 55).

Items as a Practitioner, in Order to Meet the Requirements for Telehealth:	Mean	SD
I have a feeling of dissatisfaction with the current available ways of delivering care, e.g., status quo.	2.69	1.27
I have firsthand experience of the negative effects of isolation from healthcare services.	3.84	1.18
I have a driving need to address a public or patient healthcare problem (as opposed to a practitioner specific one) that could be met by telehealth.	3.07	1.26
I am an innovator and/or champion for telehealth.	3.53	1.23
I have a sense of curiosity about the influences of telehealth on improving the delivery of health care.	3.87	0.96
I have the need to interact with other practitioners.	3.47	1.15
I have respect for others in the telehealth team.	3.98	1.05
I have examples and evidence of telehealth applications in similar contexts.	2.75	1.35
I communicate with other practitioners and the public concerning the benefits about telehealth.	2.51	1.30
I am willing to make the initial extra investment in time.	2.85	1.39
I believe telehealth can address scheduling concerns and apprehensions about overextended workloads.	3.05	1.18
I have 24 h access to telehealth equipment.	2.27	1.37
I have telehealth reimbursement plans in place.	1.67	1.02
I have dealt with apprehensions about the reliability in telehealth equipment and have good technical support and backup plans.	3.22	1.36
I have access to an established reliable and available clinical consultation network when using telehealth.	2.71	1.20
I am provided with reliable clinical content and continuing medical education through telehealth.	2.67	1.29
I attend to issues regarding liability and licensing when using telehealth.	2.75	1.32
Telehealth readiness score	3.00	0.64

**Table 4:** Scenarios on app use (unimportant = 1 to important = 5).

	Mean	SD
**Scenario 1. *Smoke-free app*** A 26-year-old woman visits your private practice/the ambulance. She tells you that she has been smoking for six years, but now wants to stop because she wants to get pregnant. You recently heard about a new smoke-free app that provides interactive support for quitting smoking. You recommend this to your patient. In your opinion, how important are the following functions of the smoke-free app for your patient?		
Support in the preparation of the smoking cessation through cost calculator and dependency tests.	3.96	1.11
Support and motivation in all phases of the behavior change process through individualized push notifications.	4.02	1.18
Instructions for relaxation exercises to relieve withdrawal symptoms.	3.89	1.13
Clear display of the progress of the smoking stop.	4.33	1.00
Representation of the positive effect on the body over time.	4.36	0.97
In your opinion, how important are the following functions of the smoke-free app for you as a treating doctor?		
View your patient´s progress electronically.	3.56	1.30
Notification of signs of relapse.	3.38	1.37
Manage control appointments, e.g., to suggest additional therapy options.	3.18	1.44
**Scenario 2. *COPD app*** A 64-year-old patient with pretreated, moderate COPD Gold II visits your private practice/the ambulance for the first time. You recently heard about a new mobile app to support the treatment of COPD. You recommend this to your patient. In your opinion, how important are the following functions of this COPD app for the patient?		
The app shows the patient how to breathe and inhale correctly with videos and breathing exercises.	4.16	1.20
The app records the prescribed medication with a barcode scanner and reminds the patient to take the medication.	3.67	1.29
The app forwards to emergency numbers in the event of acute shortness of breath.	3.89	1.13
The app records the current status of the disease every 3 months using a COPD assessment test.	3.87	1.20
The app manages appointments and reminds of control appointments.	4.05	1.10
The app records the current disease information in a disease diary.	3.51	1.25
The app uses information videos and articles to show the patient how to avoid exacerbations.	3.98	1.21
In your opinion, how important are the following functions of this COPD app for you as a treating doctor?		
View your patient’s training progress electronically.	3.55	1.21
View your patient’s therapy progress electronically.	3.60	1.26
Automatic notification of emergencies.	3.07	1.30
View the results of the regular COPD assessment tests electronically.	3.62	1.18

COPD: chronic obstructive pulmonary disease.
